# Semi-Automatic Normalization of Multitemporal Remote Images Based on Vegetative Pseudo-Invariant Features

**DOI:** 10.1371/journal.pone.0091275

**Published:** 2014-03-06

**Authors:** Luis Garcia-Torres, Juan J. Caballero-Novella, David Gómez-Candón, Ana Isabel De-Castro

**Affiliations:** Institute for Sustainable Agriculture (IAS), Spanish Council for Scientific Research (CSIC), Cordoba, Spain; NASA Jet Propulsion Laboratory, United States of America

## Abstract

A procedure to achieve the semi-automatic relative image normalization of multitemporal remote images of an agricultural scene called ARIN was developed using the following procedures: 1) defining the same parcel of selected vegetative pseudo-invariant features (VPIFs) in each multitemporal image; 2) extracting data concerning the VPIF spectral bands from each image; 3) calculating the correction factors (CFs) for each image band to fit each image band to the average value of the image series; and 4) obtaining the normalized images by linear transformation of each original image band through the corresponding CF. ARIN software was developed to semi-automatically perform the ARIN procedure. We have validated ARIN using seven GeoEye-1 satellite images taken over the same location in Southern Spain from early April to October 2010 at an interval of approximately 3 to 4 weeks. The following three VPIFs were chosen: citrus orchards (CIT), olive orchards (OLI) and poplar groves (POP). In the ARIN-normalized images, the range, standard deviation (s. d.) and root mean square error (RMSE) of the spectral bands and vegetation indices were considerably reduced compared to the original images, regardless of the VPIF or the combination of VPIFs selected for normalization, which demonstrates the method’s efficacy. The correlation coefficients between the CFs among VPIFs for any spectral band (and all bands overall) were calculated to be at least 0.85 and were significant at P = 0.95, indicating that the normalization procedure was comparably performed regardless of the VPIF chosen. ARIN method was designed only for agricultural and forestry landscapes where VPIFs can be identified.

## Introduction

Remote sensing observations are usually instantaneous and are affected by many factors, such as atmospheric conditions, sun angle, viewing angle, dynamic changes in the soil and plant–atmosphere system, and changes in the sensor calibration over time [1], [2]. The goal of radiometric corrections is to remove or compensate for all of the above effects. Exceptions to this procedure include corrections for actual changes in the ground target to retrieve surface reflectance (absolute correction) or to normalize the digital counts obtained under the different conditions and to establish them on a common scale (relative correction) [2].

Absolute radiometric corrections (ARC) make it possible to relate the digital counts in satellite image data to radiance at the surface of the Earth. This relation requires sensor calibration coefficients, an atmospheric correction algorithm and related input data among other corrections [2]. A considerable amount of research has been performed to address the problem of correcting images for atmospheric effects. Radiometric normalization of remote imagery requires all of the previously mentioned information at the time of image acquisition. For most historically remote scenes, these data are not available, and for planned acquisitions, the data may be difficult to obtain [3]. Consequently, absolute surface reflectance retrieval may not always be practical [2].

Relative radiometric normalization (RRN) based on the radiometric information intrinsic to the images themselves is an alternative whenever absolute surface radiances are not required. RRN of imagery is important for many applications, such as land cover change detection, mosaicking and tracking vegetation indices over time, and supervised and unsupervised land cover classification [2], [3], [4], [5]. Several methods have been proposed for the RRN of multitemporal images collected under different conditions at different times [2], [4]. All methods operate under the assumption that the relationship between the at-sensor radiances recorded at two different times from regions of constant reflectance is spatially homogeneous and can be approximated by linear functions. The most difficult and time-consuming aspect of these methods is the determination of suitable time-invariant features that will serve as the basis for normalization [3]. Ya’allah and Saradjian [5] developed an automatic normalization method based on regression applied to unchanged pixels in urban areas. The proposed method is based on efficient selection of unchanged pixels through image difference histogram modeling using available spectral bands and calculation of relevant coefficients for dark, gray and bright pixels in each band. Yang and Lo [6] studied five methods of RRN applied to Landsat images. This method includes pseudo-invariant features, radiometric control set, image regression, no-change set determined from scattergrams, and histogram matching, all of which require the use of a reference-subject image pair. Factors that affect the performance of RRN include land-use/land-cover distribution, water-land proportion and topographic relief [6]. Ground reference data are expensive and difficult to acquire for most remotely sensed (satellite) images, and the selection of PIF is generally subjective [2]. In practice, vegetative targets of absolutely constant reflectance do not exist. Therefore, the concept of PIF is adopted with the assumption that the reflectance is constant over time [2].

Several authors have developed powerful statistical approaches to determine invariant features for the atmospheric normalization of image pairs. Hall et al. (1991) [7] and Coppin and Bauwer (2004) [8] developed radiometric rectification techniques for land cover change detection through the use of landscape elements whose reflectance values are nearly constant over time. Hall et al. (1991) [7] selected PIFs with two sets of data, bright and dark. The two sets were selected in different images by visual inspection. Du et al. (2002) [2] developed a new procedure for radiometric normalization between multitemporal images of the same area. In this method, the selection of PIF is performed statistically, and quality control and principal component analysis (PCA) are used to find linear relationships between temporal images of the same area. Several authors have proposed a change-detection technique called multivariate alteration detection (MAD), which is invariant to linear and affine scaling [3], [9]. Thus, if MAD was used for change detection applications, pre-processing by linear radiometric normalization is superfluous. An iteratively re-weighted modification of the MAD transformation (IR-MAD) established a better background of no change upon which significant changes can be examined [10]–[11]. Some authors have used the IR-MAD transformation for relative radiometric normalization of multitemporal images and MAD transformation for change detection [12]. Others converted digital number values to reflectance directly by relative radiometric normalization using IR-MAD [13]. Baisantry et al. (2012; [14]) performed RRN using MAD transformation and selected PIFs automatically through the Bin-Division Method. Kim et al. (2012; [15]) developed a method designed to automatically extract pseudo-invariant features for the RRN of hyperion hyperspectral images and used band-to-band linear regression. Philpot and Ansty (2013; [16]) developed an analytical formula that relates pseudo-invariant features (PIFs) to the radiometric properties of the scenes. The formula is then inverted to yield an estimate of the ratio of the transmission spectra of the two images given the path radiance for each scene and a set of invariant features. Sadeghi et al. (2013) [17] proposed an automated RRN to adjust a non-linear based on artificial neural network and unchanged pixels.

QUAC (quick atmospheric correction) and FLAASH (fast line-of-sight atmospheric analysis of spectral hypercubes) are atmospheric correction modules used in ENVI image processing software (Exelis-Visual Information Solutions, Inc. 4990 Pearl East Circle Boulder, CO 80301 USA, http://www.exelisvis.com). QUAC is an on-the-fly method for use in real-time data processing that determines parameters directly from the information contained in the scene using the observed pixel spectra [18]. FLAASH is a physics-based correction method built on MODTRAN4 atmospheric correction software [19]. FLAASH allows the user to define all parameters that influence atmospheric absorption and scattering, such as relative solar position, atmospheric, aerosol, and scattering models, and visibility parameters, among others. The advantage of QUAC is that an in-scene approach is easily implemented, while FLAASH uses a very diverse atmospheric ancillary parameter, and the data are therefore highly tunable by the image expert. Hu et al. (2011, [20]) compared the FLAASH and MAD normalization methods on Landsat time-series images. Other authors applied FLAASH to change detection applications [21].

To our knowledge, no information is available on relative image normalization (ARIN) of multitemporal agricultural scenes based on VPIFs or on the development of software to achieve this semi-automatically. Our specific objectives were as follows: 1) to describe the ARIN procedure and implement it in the GeoEye-1 multitemporal image series; 2) to comparatively study the selected VPIFs in relation to the ARIN method efficacy; 3) to compare ARIN-transformed multitemporal images to the original (ORI) and to the FLAASH and QUAC calibrated images; and 4) to develop semi-automatic software to normalize any set of multitemporal remote imagery by identifying VPIFs.

## Materials and Methods

### 1. ARIN Procedure and Software

The procedure developed for the relative normalization of multitemporal images consists of the following steps: 1) selecting one or several VPIFs; 2) defining the same parcel or parcels for each selected VPIF in each multitemporal image; 3) extracting the VPIF spectral band data for each image; 4) calculating the correction factors (CFs) for each image band to fit each band value to the average value of the image series; and 5) obtaining the normalized images by transforming each band through CF linear functions. Further information of VPIF and of vegetative variant features (VVF) will be given later in this article. Basically they coincide with permanent orchards/mature tree plantations and annual/herbaceous crops, respectively. We select the VPIF parcels at random, among many parcels of very similar characteristics available in our agricultural scene. Main steps of ARIN procedure can be achieved by conventional image processing menus including the parcel definition, the spectral band data extraction, and the image liner transformation through the estimated correction factors (CF), as will be later defined. The CF can be calculate manually or in a excel sheet once the VPIF spectral band values has been extracted.

Environment for Visualizing Images (ENVI 4.8 and ENVI 5.0, Exelis-Visual Information Solutions) software was used to visualize and process the images. Generally, mature non-deciduous tree orchards and permanent lawn green cover are eligible to be VPIFs, and at least one must be present in the scene for normalization. A parcel of the selected VPIF needs to be drawn through the ROI/SHAPE menu in one image and then moved to the rest of the image series through the VECTOR/SHAPE menu (convert the ROI to a DXF vector). The B, G, R and NIR spectral bands of the selected VPIF can be extracted for each image through the ROI/SHAPE Tool Statistical menu.

**Figure 1:**
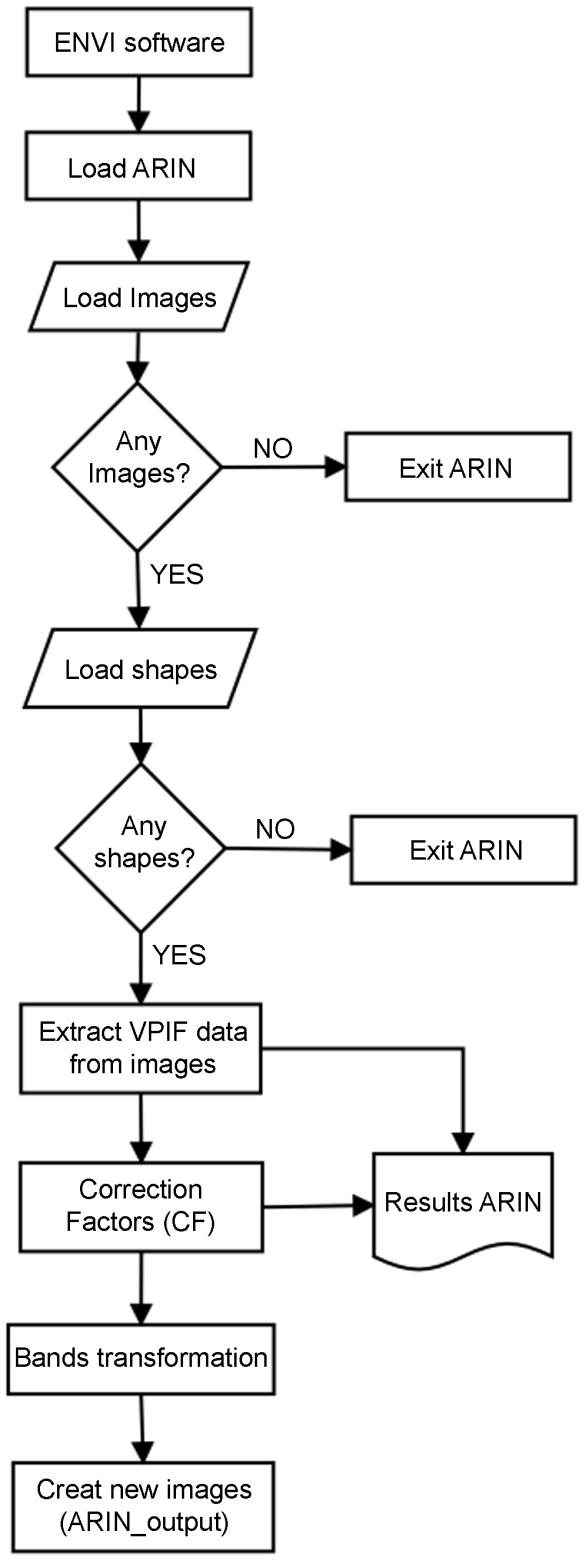
ARIN flowchart.

**Figure 2:**
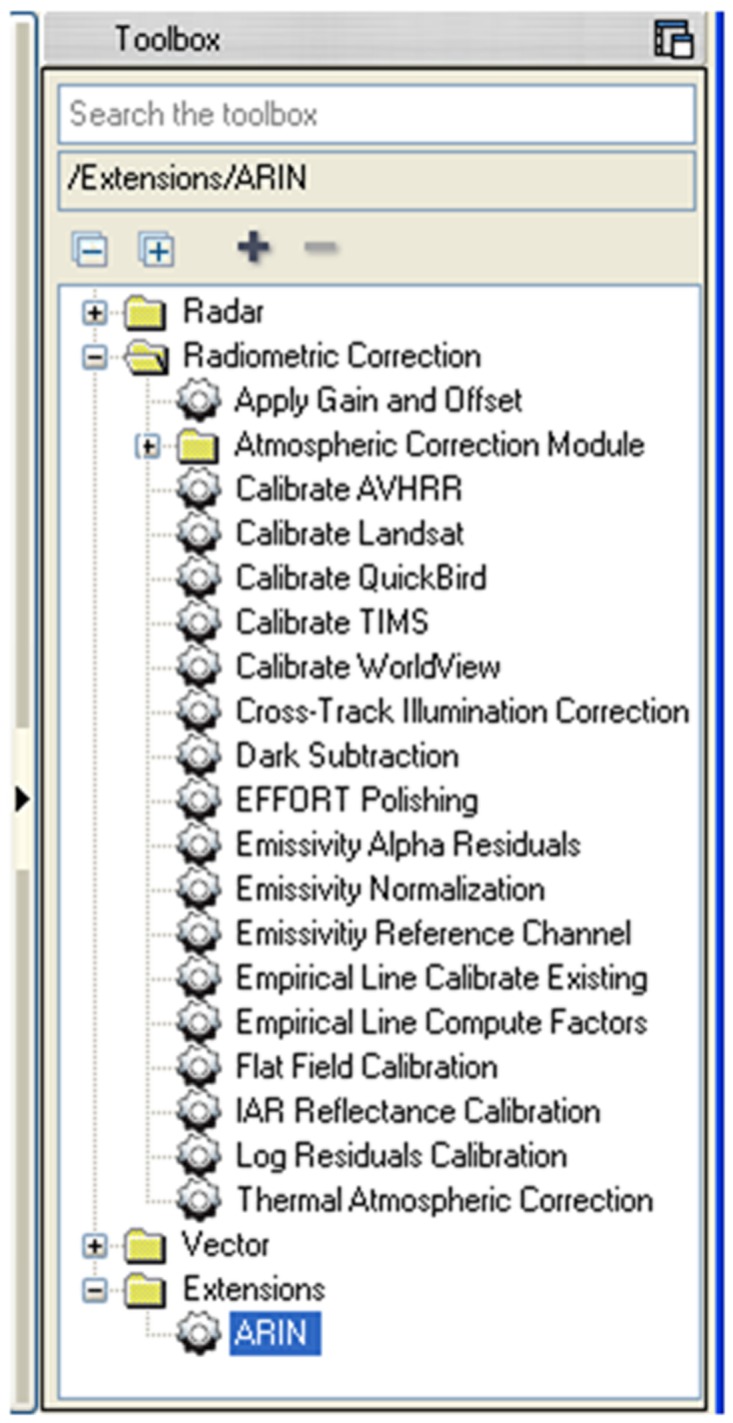
View of radiometric correction available in ENVI5.0 and ARIN software as an extension.

**Figure 3:**
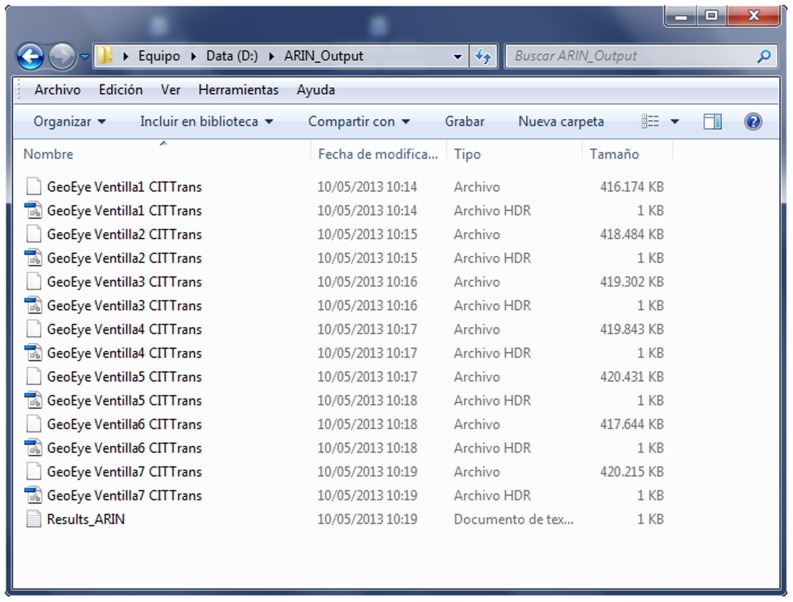
ARIN_output: normalized images (“transf”) using CIT VPIF.

**Figure 4:**
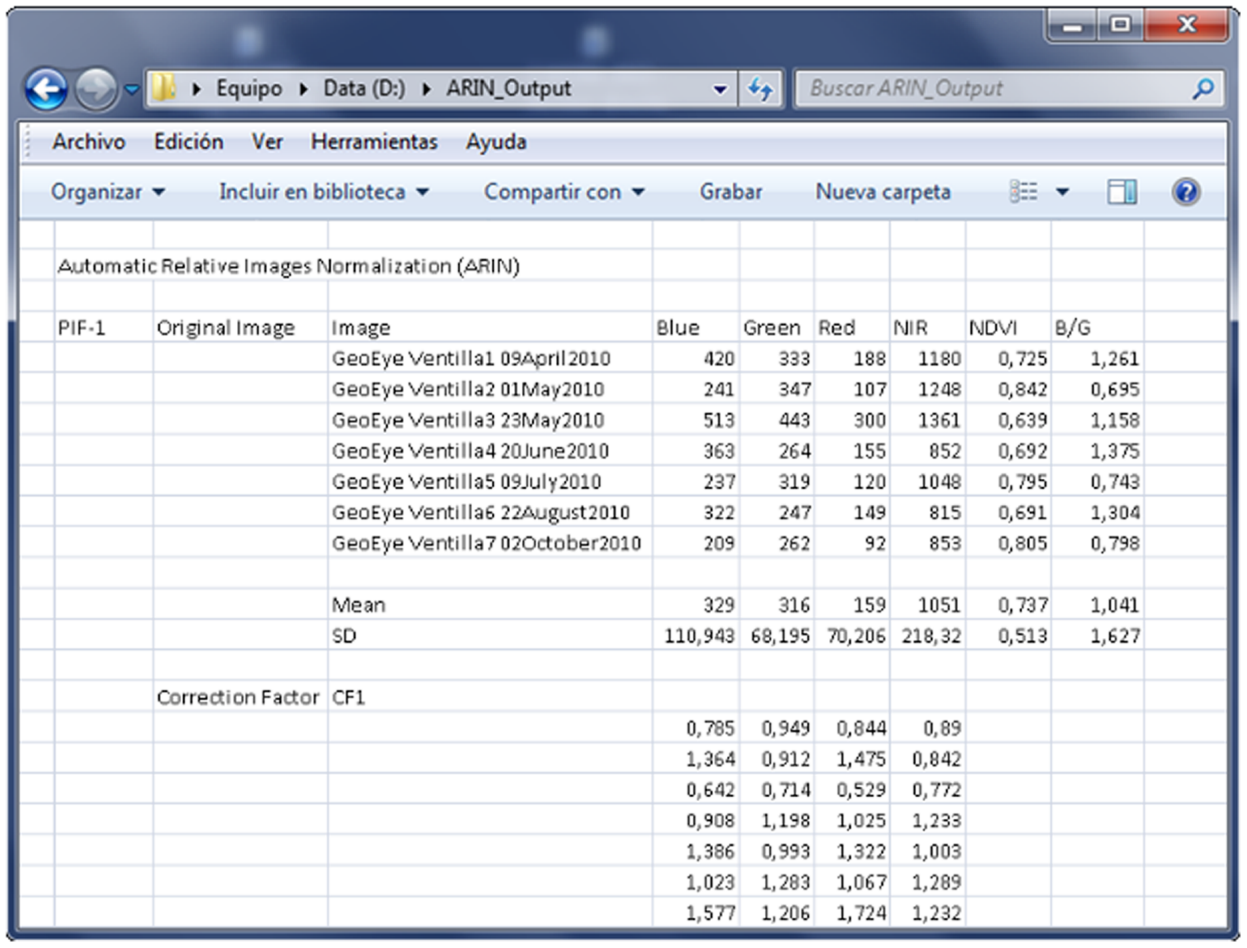
ARIN_results: transposed.txt and Excel files. The VPIF spectral bands of the original images and the corresponding band correction factors are shown.

**Figure 5:**
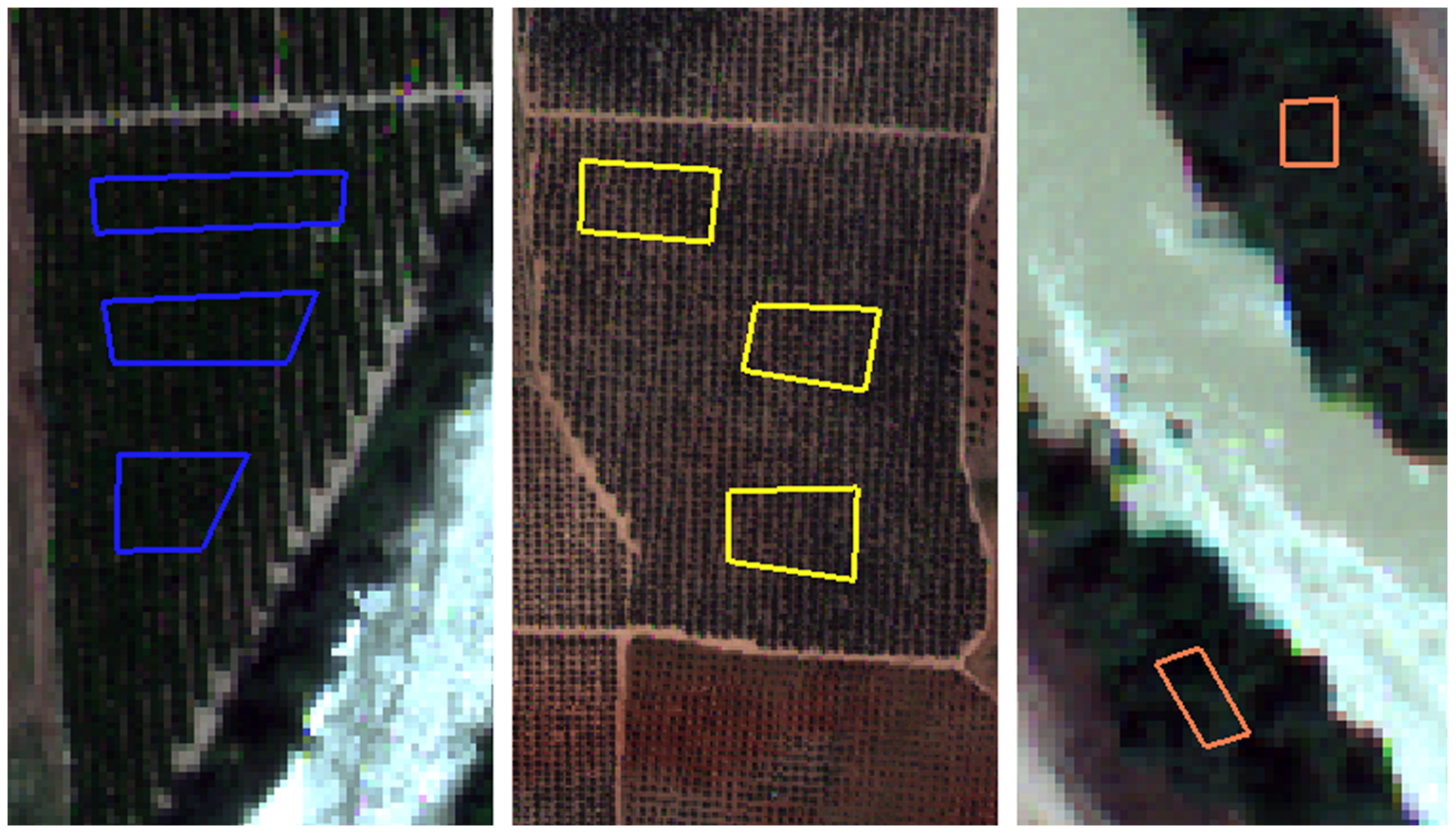
Partial view of the vegetative pseudo-invariant features (VPIFs) used in this study. a) CIT-citrus, b) OLI-olives, and c) POP-poplar groves. The VPIF SHP files are transposed to other multi-temporal images during the ARIN process.

The CFs of any VPIF spectral band, for example, the G band of the image i (CF_Gi_), are defined as the ratio G_m_/G_i_, where G_m_ is the average original value of the G band in the original image (ORI), and G_i_ is the band value of image i. Then, each band of each ORI will be transformed by applying the corresponding linear CF through the Basic Tool-Band Math menu. The normalized image will be composed of the transformed bands through the Layer Stacking menu. To semi-automatically perform the previously described steps (steps 2 through 5), the so-called ARIN software and procedure were developed [22], [23]. The ARIN flowchart is shown in Figure 1. The main ARIN software screens are shown in Figures 2, 3, and 4. A partial view of the vegetative pseudo-invariant features (VPIFs) used in this study is shown in Figure 5.

### 2. ARIN Procedure Validation

#### 2.1 Study location and GeoEye-1 images series

Seven multi-spectral and panchromatic GeoEye-1 satellite images (GeoEye-1, 2012), each covering approximately 100 km^2^, were taken over the same area of LaVentilla village (a province of Cordoba, southern Spain) from April to October 2010. The geographic coordinates (Universe Transverse Mercator System, Zone 30 North) in the upper-left corner of the images were X = 315206 m/Y = 4186133 m. The images were taken on April 9, May 1, May 23, June 20, July 9, August 22 and October 2 and are named V1 to V7, respectively. The panchromatic image was 0.50 m pixels^−1^, and the multi-spectral-image spatial resolution was 2.00 m pixels^−1^, providing information on blue (B, 450–510 nm), green (G, 510–580 nm), red (R, 655–690 nm) and near-infrared (NIR, 780–920 nm) spectral bands. The swath width was 15.2 km. The ground was predominantly flat, with an average slope grade of 2.12%. The georeferencing accuracy of the GeoEye-1 images was improved by using ground control points (GCPs) and image-to-image co-registration [24].

#### 2.2 Land uses and selected variant and invariant vegetative features

The LaVentilla area was surveyed approximately every 3 weeks from April to October 2010 to identify the crop of each parcel, its stage of development, and any key agricultural features. A total of 23 land uses were identified in the Geo-Eyes-1 scenes. Vegetative systems, such as alfalfa, avena, broad beans, citrus orchards, chickpeas, corn, cotton, Mediterranean forest, olive orchards, potatoes, sunflower, rapeseed, poplar groves and winter wheat, among others, were identified. Additionally, non-vegetative land uses, such as rivers, water reservoirs, paved roads, bare soil roads, and civil buildings, were also found. The phenotypes of some herbaceous vegetation parcels, such as wheat, sunflower, corn and cotton, varied considerably throughout the growing season and can thus be designated variant vegetative features (VVFs). The phenotypes of high density adult tree plantation parcels, such as citrus orchards (CIT), olive orchards (OLI), and poplar groves (POP), demonstrate much less vegetation change throughout the cropping season. Thus, they can be designated pseudo-invariant vegetative features (VPIFs). To show the differences between VVF and VPIF parcels, the spectral bands and NDVI vegetative index evolution were determined in four parcels of approximately 0.3–0.5 ha for each selected VPIF (CIT, OLI and POP) and for wheat (WHT) and corn (CRN), as representatives of the winter (autumn-sown) and summer (spring-sown) VVFs.

#### 2.3 Implementation of the ARIN procedure

CIT, OLI and POP were used as VPIFs. Four parcels of approximately 0.3–0.5 ha were drawn for each VPIF using the regions of interest (ROI-VECTOR)/SHAPE menu of ENVI. The ARIN procedure provided the B, G, R and NIR spectral bands, the NDVI and the G/B vegetative indices for the original and transformed/normalized images. Normalization was achieved using a single VPIF and by validating the other two VPIFs or by using two VPIFs consecutively and validating with the other.

#### 2.4. Absolute corrections using QUAC and FLAASH

To allow comparison with ARIN, the original V1 to V7 images were transformed using QUAC and FLAASH software. QUAC software requires the presence of dark and bright pixels in the images to serve as a basis for the implemented corrections. FLAASH was implemented by fitting to GeoEye-1 sensor specifications and to the atmospheric mid-latitude summer geographic area where the satellite images were taken. This area was aerosol rural, the highest image ground elevation was 150 m, the water column retrieval parameter was 2.92, and the scene visibility parameter was 100 km for all images except for the V7 image (140 km).

#### 2.5. Statistical parameters

VPIF and VVF parcels spectral band and vegetation index data were subjected to analysis of variance and means were separated at the 5% level of significance by the least significance difference (LSD) test with the use of SPSS Statistical-21 software (IBM North America, New York, NY, United States). For any original or transformed image, the VPIF mean, range, standard deviation (s. d.) and root mean standard error (RMSE) ofthe band spectral values, NDVI and B/G vegetation indices were determined. The root mean square error (RMSE) of the series of images was calculated by the following equation [25]:
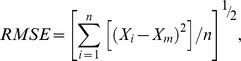
Where n is the number of images, X_i_ is the values of each image and X_m_ is the average of all images. The smaller range, s. d. and RMSE of data in a given series of images indicated more uniformity among images than in the others.

The correlation coefficients and the level of significance between CFs were determined across VPIFs for each image band and for all images.

## Results and Comments

### 1. Evolution of Vegetative Variant and Pseudo-invariant Features

**Figure 6:**
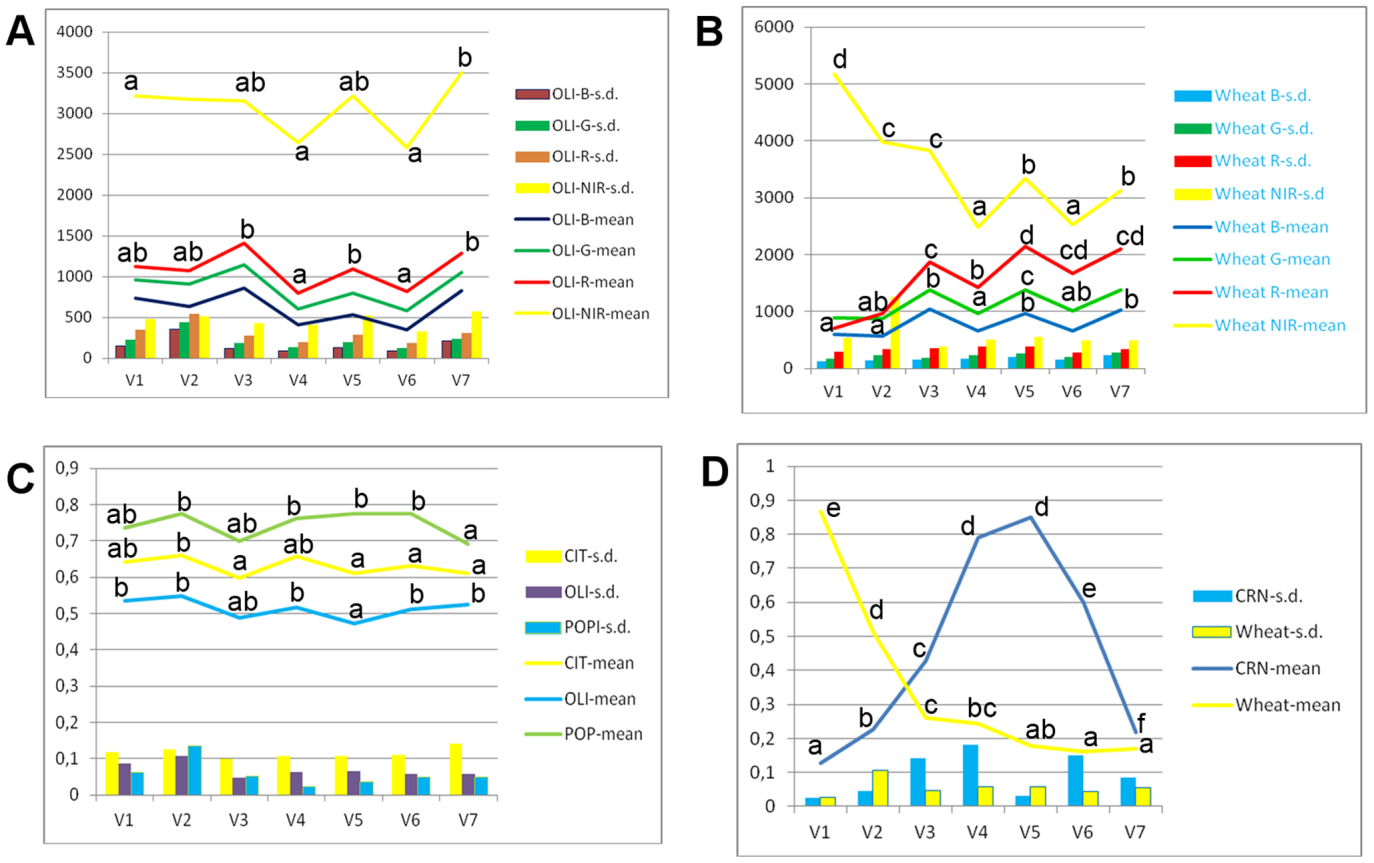
a) and b) Spectral band evolution in pseudo-invariant (OLI, olive) and variant (wheat) features; c) and d) NDVI evolution in pseudo-invariant features (CIT, citrus; OLI, olives, and POP, poplar groves); and d) winter wheat and corn as representative cropping systems that have variant vegetative features. The abscissa is the remote image timing (V1- early April to V7 - early October, interval of approximately one month; data are presented as the means, and vertical bars represent the standard deviation (s. d.) of six parcels of approximately 3 ha each).

**Figure 7:**
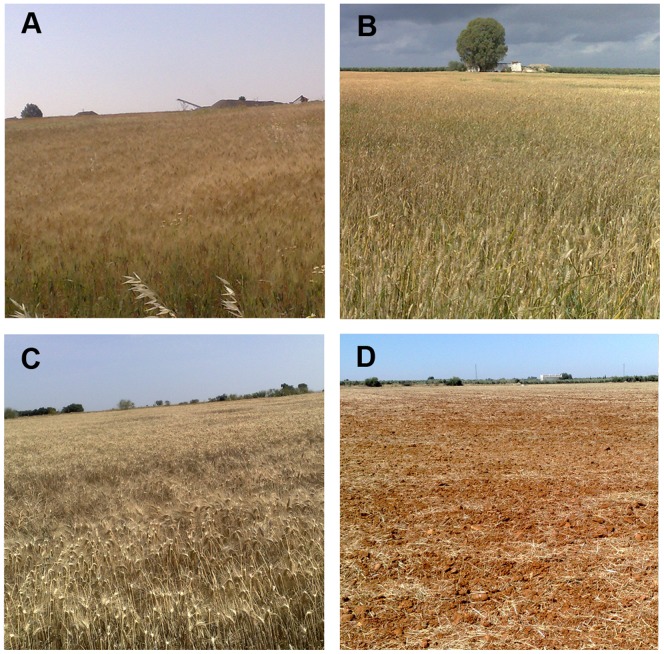
Wheat crop evolution. A: early April, grain filling growth stage, showing intense green color and therefore high NDVI values; B: early May, mid-senescence, green-yellowish color, and mid-NDVI values; C: late May/early June, late senescence, predominant yellow color, low NDVI values; D: stubble, typical of mid-June throughout the summer. These growth stages roughly coincide with the V1, V2, V3 and V5 satellite images taken, respectively, and with the wheat NDVI data evolution shown in Figure 6d.

**Figure 8:**
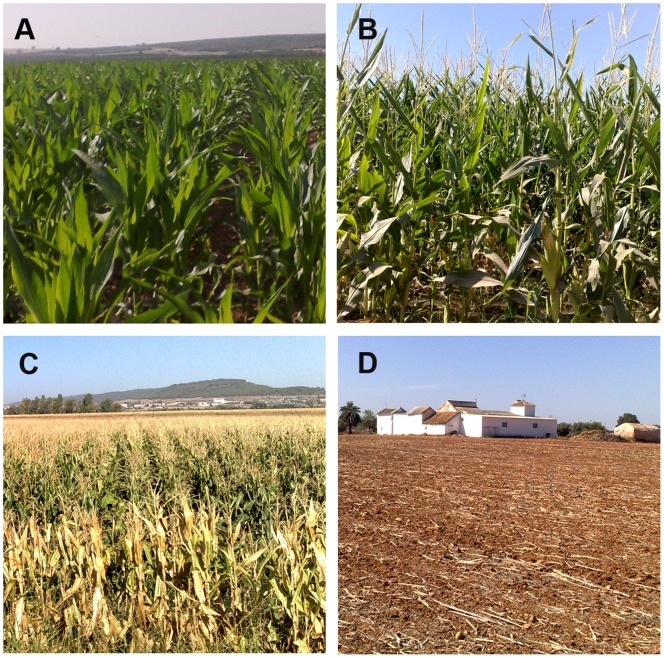
Corn crop evolution. A: late May and early June, corn at the vegetative growing phase, characterize with increasing NDVI values, and coinciding with the V3–V4 satellite images of this stage; B: flowering stage, July, V5 image; C: senescence period, which take place in the second part of August (NDVI values decrease; satellite image V6); and D: corn stubble, beyond mid-September (satellite image V7). Corn NDVI data evolution shown in Figure 6d.

**Figure 9:**
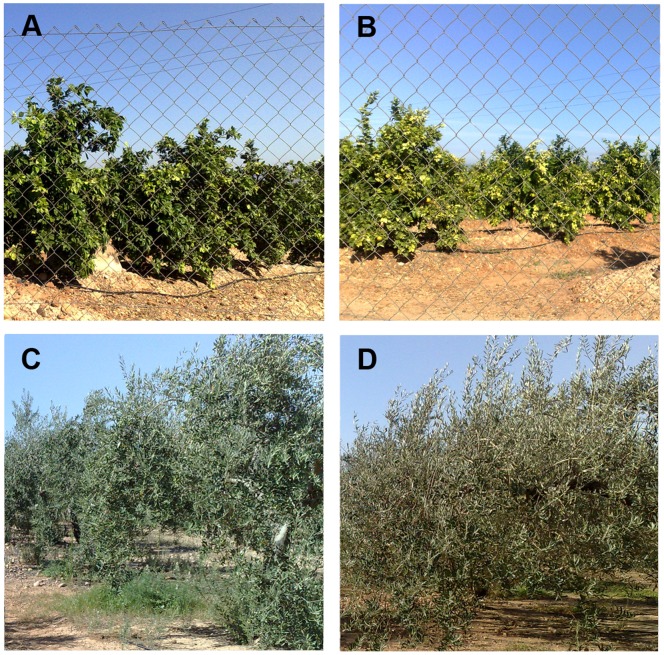
Canopy structure of citrus (A and B) and olive (C and D) orchards vary very little throughout the year and particularly in the main growing season (April to October). Photographs A and C were taken early May and B and D late September, roughly coinciding with the V2 and V7 satellite images of this study. In photograph D can be appreciated the orange fruit but not the olives in photograph D at the distance where the photograph was taken. Due to the little changes in the trees canopy and soil surface covered the NDVI values of citrus and olive are very similar throughout the growing season (Figure 6d), and can be used as pseudo-invariant features for radiometric normalization as shown in this study.

Generally, the evolution of the spectral bands and the NDVI throughout the growing season varied to a greater extent in VVFs than in VPIFs (Figures 6, 7, 8, and 9). For example, the NIR band average, range and s. d. for OLI were 466, 240, and 81 (Figure 6a), respectively, whereas for WHT, these values were 3494, 2683 and 936, respectively (Figure 6b), for the images series. Similarly, the NDVI evolution varied significantly in VVFs, while it was relatively stable in VPIFs. For example, the NDVI mean, range and s. d. were 0.63, 0.064 and 0.024 for CIT and 0.34, 0.70 and 0.26 for WHT, respectively (Figure 6c & d). These data confirmed that the phenotype, morphology, development, and observable physical characteristics of perennial plantation are very stable throughout the agricultural season for VPIFs (Figure 9), while the opposite is true for the herbaceous cropping systems (VVFs, Figures 7 and 8). This observation has been obvious to field workers and agronomists for many years. This simple, evident finding is very important for land use classification of agricultural scenes through remote sensing.

### 2. Relative Radiometric Normalization

#### 2.1 Using a single VPIF

The spectral band values of the original GeoEye-1 image series for the given VPIFs varied considerably among the images (Table 1). For example, the CIT B band digital values for the images V2 and V6 were 241 and 322, respectively, and the POP NIR values for the same images were 995 and 785, respectively (Table 1). Consequently, the vegetation indices varied considerably among the original images at any given VPIF (Table 2). For example, the CIT NDVI values varied from 0.64 at V3 to 0.81 at V7, and the POP NDVI values ranged from 0.62 at V3 to 0.81 at V2. This wide variation in the VPIF band digital counts and vegetation indices among images indicates that the radiometric normalization process is highly recommended for a comparative follow-up of cropping systems and other environmental features in any multitemporal image series.

**Table 1:** Vegetative pseudo-invariant feature (VPIF) spectral band values of the original (ORIG) and VPIF ARIN-transformed (-transf.) images.

			Series of images^1^							Statistical			
VPIF	Spectral Band	ImagesType	V1	V2	V3	V4	V5	V6	V7	Mean	Range	S. d.^2^	RMSE
CIT^1^	B	ORIG^2^	420e^2^	241b	513f	363b	237a	322d	209a	329 g^3^	304 g	111 g	103 g
		POP-transf.	334b	335b	347b	310ab	341b	324b	307a	328 g	40 h	15 h	14 h
		OLI-transf.	343ab	322a	346b	320a	330a	338ab	283a	326 g	63 h	21 h	20 h
	G	ORIG	333c	347c	443d	264ab	319c	247a	262b	316 g	196 g	68 g	63 g
		**P**OP-transf.	322bc	346cd	350cd	274a	324bc	302ab	281a	314 g	76 h	30 h	28 h
		OLI-transf.	342d	323bc	352cd	289ab	309bc	323bc	266a	315 g	86 h	30 h	28 h
	R	ORIG	188c	107b	300e	155cd	120a	149c	92a	159 g	208 g	70 g	65 g
		**P**OP-transf.	158ab	158ab	197b	133a	175ab	142a	129a	156 g	68 h	24 h	23i
		OLI-transf	163ab	136a	190b	140a	153a	163ab	135a	154 g	55 h	20 h	19 h
	NIR	ORIG	1180c	1248d	1361e	852a	1048b	815a	853a	1051 g	546 g	218 g	202 g
		**P**OP-transf.	1173d	1095cd	1192d	904a	1005b	906a	1031bc	1048 g	298 h	117 h	108 h
		OLI-transf.	1247d	1120d	1088cd	951ab	1000bc	1029bc	895a	1047 g	342 h	117 h	108 h
OLI	B	ORIG	462e	283b	559f	428d	271a	360c	278b	377	288 g	110 g	102
		**P**OP-transf.	367a	393b	378ab	365a	390ab	362a	409b	381	47 h	17i	17 h
		CIT-transf.	362a	386c	359a	388bc	376ab	368ab	438c	382	79i	27 h	26 h
	G	ORIG	349c	384d	450e	327a	368c	273a	352c	358	177 g	54 g	50 g
		**P**OP-transf.	337a	383bc	355ab	340a	375bc	335a	377bc	357	48 h	21 h	19 h
		CIT-transf.	331ab	350bc	321a	392c	366b	351b	425d	362	104i	36i	34i
	R	ORIG	312d	214b	427e	300	212ab	248c	185a	271	242 g	83 g	77 g
		**P**OP-transf.	261ab	314c	281b	259ab	309c	237c	259ab	274b	77 h	28 h	27 h
		CIT-transf.	263a	316c	226a	308c	280ab	264a	319c	282	93 h	34 h	34 h
	NIR	ORIG	731c	861d	966e	692b	809c	612a	736bc	772	354 g	117 g	108 g
		**P**OP-transf.	727b	756b	846c	735ab	776b	680a	890c	773	210 h	72 h	67 h
		CIT-transf.	651a	725a	746a	854b	812b	789a	907b	783	256 h	85 h	80 h
POP	B	ORIG	428c	245b	504d	399c	237a	339b	231a	340	273 g	108 g	100 g
		CIT-transf.	336ab	335ab	324a	362ab	329a	346ab	365b	342	41 h	16 h	15 h
	.	OLI-transf.	350ab	327ab	340ab	352ab	330ab	355b	314a	338	41 h	15 h	14 h
	G	ORIG	307c	298cd	376d	286bc	292bc	243ac	277b	297	133 g	40 g	37 g
		CIT-transf.	292a	272a	268a	342a	290a	311a	335a	301	74 h	29 h	27 h
		OLI-transf.	315ab	277a	299ab	313ab	283ab	317b	282ab	298	40i	17i	16i
	R	ORIG	186bc	106a	237d	181b	107a	163b	111a	156 g	131 g	50 g	46 g
		CIT-transf.	157ab	157ab	125a	186ab	141ab	174ab	192b	162 g	67 h	24 h	23 h
		OLI-transf.	162a	135a	151a	164a	137a	179a	163a	156 g	44 h	16i	15i
	NIR	ORIG	879b	995b	997b	823ab	911b	785ab	722a	873 g	275 g	104 g	96 g
		CIT-transf.	782a	838a	770a	1015b	913ab	1013b	890a	889 g	245 g	100 g	94 g
		OLI-transf.	928b	893ab	797a	918b	869ab	992b	758a	879 g	234 g	80 g	74i

1 Series of images: from V1, early April, to V7, October.

2 Abbreviations: ORI; original images; CIT, citrus orchards; OLI, Olive orchards; POP, poplars grove; -transf., transformed images; B, blue; G, Green; R, red; NIR, near infra-red; S. d., standard deviation; RMSE, Root Mean Square Error.

3 For each VPIF, spectral band and image type the data of the multitemporal images followed by the same letter are not significantly different at P≥0.05.

4 For each VPIF and spectral band statistical data of image types followed by a different letter are significantly different at P≥0.05.

**Table 2:** Selected vegetative pseudo-invariant feature (VPIF) vegetation indices of the original (ORIG) and VPIF ARIN-, QUAC- and FLAASH–transformed (-transf.) images.

			Series of images^1^							Statistical			
VPIF	VegetationIndex	Images	V1	V2	V3	V4	V5	V6	V7	Mean	Range	S. d.^2^	RMSE.^2^
CIT	NDVI	ORIG^2^	0.73b^3^	0.84d	0.64a	0.69a	0.79c	0.69a	0.81cd	0.74 g^4^	0.20 h	0.07 h	0.07i
		OLI+POP-transf.	0.77ab	0.75a	0.72a	0.75a	0.71a	0.73a	0.78b	0.74 g	0.08 g	0.03 g	0.02 g
		QUAC-transf.	0.79a	0.85ab	0.79a	0.90a	0.79a	0.90b	0.89ab	0.84 h	0.12 g	0.05 g h	0.05 hi
		FLAASH-transf.	0.76b	0.69a	0.80c	0.74a	0.78b	0.78b	0.79bc	0.76 g	0.11 g	0.04 g	0.07i
	B/G	ORIG	1.26dc	0.69a	1.16c	1.38d	0.74a	1.30d	0.80b	1.05 h	0.68i	0.29i	0.45i
		OLI+POP-transf	1.02a	0.96a	0.98a	1.12a	1.04a	1.06a	1.08a	1.04 h	0.16 g	0.06 g	0.06 g
		QUAC-transf	0.44b	0.57c	0.24a	0.43b	0.54c	0.43b	0.47bc	0.45 g	0.33 h	0.11 h	0.17 g
		FLAASH-transf.	0.59b	0.64c	0.55b	0.59bc	0.47a	0.64c	0.66c	0.59 g	0.19 g	0.07 g	0.27 h
OLI	NDVI	ORIG	0.40a	0.60c	0.39a	0.40a	0.58c	0.42a	0.60c	0.48 g	0.21 h	0.10 h	0.09 h
		CIT+POP-transf.	0.46ab	0.40a	0.49b	0.47ab	0.42a	0.48bc	0.54c	0.47 g	0.14 g	0.05 g	0.04 g
		QUAC-transf.	0.37a	0.51bc	0.54c	0.60cd	0.45b	0.70d	0.66d	0.55 g	0.33	0.11 h	0.10 h
		FLAASH-transf.	0.50b	0.42a	0.45a	0.42a	0.49b	0.45a	0.45a	0.45 g	0.08 g	0.03 g	0.04 g
	B/G	ORIG	1.32c	0.74a	1.24c	1.31c	0.74a	1.32c	0.79b	1.07 g	0.59i	0.29i	0.271
		CIT+POP-transf.	1.08a	1.02a	1.05a	1.06a	1.03a	1.07a	1.07a	1.05 g	0.06 g	0.02 g	0.02 g
		QUAC-transf.	0.60c	0.76d	0.33a	0.60b	0.63c	0.56b	0.67d	0.59 g	0.42i	0.13 h	0.12 h
		FLAASH-transf.	0.54a	0.76c	0.68b	0.68b	0.61ab	0.82c	0.79c	0.70 g	0.28 h	0.10 h	0.09 h
POP	NDVI	ORIG	0.65ab	0.81d	0.62a	0.64a	0.79d	0.66b	0.73c	0.70 g	0.19 h	0.08 h	0.07 h
		CIT+OLI-transf.	0.70ab	0.73b	0.67a	0.69ab	0.72b	0.69ab	0.64a	0.69 g	0.09 g	0.03 g	0.03 g
		QUAC-transf.	0.73a	0.81b	0.81b	0.85c	0.80ab	0.88c	0.81b	0.81 g	0.15 h	0.05 g h	0.04 g
		FLAASH-transf.	0.70ab	0.70ab	0.75b	0.76b	0.75b	0.67a	0.76b	0.73 g	0.09 g	0.03 g	0.03 h
	B/G	ORIG	1.39b	0.82a	1.34b	1.40b	0.81a	1.40b	0.83a	1.14 h	0.58 i	0.30 i	0.27i
		CIT+OLI-transf.	1.11a	1.18a	1.14a	1.13a	1.16a	1.12a	1.12a	1.14 h	0.07 g	0.03 g	0.02 g
		QUAC-transf.	0.60b	0.82c	0.33a	0.63b	0.68b	0.58b	0.64b	0.61 g	0.49i	0.15 h	0.14 h
		FLAASH-transf.	0.72c	0.79c	0.67b	0.67b	0.59a	0.78c	0.77c	0.71 g	0.20 h	0.07 g	0.08 h

1 Series of multitemporal images: from V1, early April, to V7, October.

2 Abbreviations: ORI; original images; CIT, citrus orchards; OLI, Olive orchards; POP, poplars grove; -transf., transformed images; S. d., standard deviation; RMSE, Root Mean Square Error. Vegetation indexes: NDVI: (NIR−R)/NIR+R); B/: B and G are spectral bands.

3 For each VPIF, vegetation index and image type the data followed by the same letter are not significantly different at P≥0.05.

4 For each VPIF and vegetation index statistical data of image types followed by a different letter are significantly different at P≥0.05.

Generally, the range and s. d. of the spectral bands and vegetation indices in the ARIN-transformed images were considerably reduced compared to those of the original images (Table 1 and 2), regardless the VPIF considered. First, it should be noted that for a single transformation (using just one VPIF), the spectral bands and vegetation indices of the VPIF taken as reference produce exactly the same value for any transformed image. This value is the average of the image series, for example, 329 and 1051 for the CIT B and NIR spectral bands, respectively. Additionally, the corresponding range and s. d. are negligible.

The ARIN process was an efficient normalization process regardless of the single VPIF or the combination of VPIFs chosen. The selection of the single VPIF used for the ARIN normalization process only slightly affected the normalization results of other VPIFs. For example, the range, s. d. and RMSE of the CIT VPIF B and NIR bands at the POP transformed images series were 40, 15 and 14 and 288, 117 and 108, respectively, whereas these values were 304, 111 and 103 and 546, 218 and 202 in the original images (Table 1). Similarly, the range, s. d. and RMSE values for the R band of the CIT VPIF in the OLI transformed images were 55, 20 and 19, compared to 208, 70 and 65 for the original images, respectively (Table 1).

#### 2.2 Using two VPIFs consecutively

For each selected VPIF, the normalization effect caused by the ARIN procedure was also determined using two VPIFs consecutively; thus, the potential slight stationary phenotypic variation could be balanced for each VPIF. Applying the ARIN process using two VPIFs consecutively is also an effective method to normalize the multitemporal series of images. For example, after normalization with the pseudo-invariants OLI+POP, the CIT NDVI range, s. d. and RMSE of the image series were 0.08, 0.03 and 0.02, respectively, in comparison to 0.20, 0.07 and 0.07for the original images (Table 2). Similarly, the consecutive implementation of ARIN CIT+POP resulted in OLI NDVI range, s. d. and RMSE values of 0.14, 0.05 and 0.04 in comparison to 0.21, 0.10 and 0.09 for the original images, respectively.

#### 2.3 VPIF correlation factors

The VPIF spectral band CFs used to implement the ARIN linear normalization procedure varied greatly among the spectral bands for any given image (Figure 4) and among images for any given spectral band. Furthermore, the correlation coefficients between the CFs among the VPIFs for any spectral band and for all bands were found to be at least 0.85 and were significant at P = 0.95 or higher (Table 3). This finding also demonstrates that the ARIN normalization process is efficient regardless of the VPIF selected.

**Table 3:** Correlation coefficients between the spectral band CFs of VPIF CIT, OLI and POP.

	Spectral band				
VPIF^1^	B	G	R	NIR	Overall
CIT vs. OLI	0.97**	1.0**	0.97**	0.85*	0.96**
CIT vs. POP	0.98**	0.91*	0.92*	0.89*	0.93**
OLI vs. POP	0.99**	0.92*	0.93**	0.85*	0.92**
Overall	0.97**	0.93**	0.92**	0.87**	0.93**

1 Abbreviations: VPIF; vegetative pseudo-invariant features; CIT, citrus orchards; OLI, olive orchards; POP, poplar groves; B, blue; G, green, R, read, NIR, near-infrared; * and ** Statistically significant at ≥95% and ≥99% probabilities.

### 3. ARIN vs. QUAC and FLAASH

Generally, for the series of GeoEye-1 images studied, ARIN was more efficient than QUAC and as efficient as FLAASH, varying slightly with the VPIF selected. For example, if we consider the VPIF CIT, the NDVI s. d. of the original, OLI+POP-transf., QUAC and FLAASH images were 0.07, 0.03, 0.05 and 0.08, respectively, and the same statistics for the B/G index were 0.29, 0.06, 0.11 and 0.07 (Table 2). Considering the VPIF POP and the s. d. and RMSE statistical, the results are better for ARIN CIT+OLI-transf. (0.03 and 0.03) and FLAASH (0.03 and 0.03), followed by QUAC (0.05 and 0.04) compared to the original images (0.08 and 0.07) (Table 2). Considering the B/G index, the ARIN-transf. procedure was also more effective than QUAC and FLAASH in any VPIF studied.

## Discussion

In our study, the VPIF spectral band and vegetation index values of the original GeoEye-1 images varied considerably among the images, indicating that the calibration or radiometric normalization process is highly recommended for comparative follow-up of cropping systems. In fact, the calibration and normalization of multitemporal images has been a challenge in remote sensing for decades [1]–[16].

ARC relates image digital counts to radiance at the surface of the Earth and requires sensor calibration coefficients, an atmospheric correction algorithm and related input data, among other corrections [2]. For most historical images, such data are not available, and for planned acquisitions, they may be difficult to obtain [3]. Consequently, ARC retrieval is not often a practical method [2]. RRN is based on the radiometric information intrinsic to the images themselves and is an alternative whenever absolute surface radiances are not required, as in change detection applications or for supervised land cover classification [3], [4].

To our knowledge, no RRN methods for multitemporal remote images using VPIF as a reference have previously been developed. The concept of PIF is adopted with the assumption that the reflectance is constant over time [2]. Moreover, any individual plant, cropping system, or vegetative feature varies with time, and therefore, there is a unanimous agreement that the reflectance is not an absolute invariant. As we have shown, most agricultural areas have vegetative parcels that change drastically throughout the growing season, such as annual herbaceous crops (VVFs), while others features, such as dense forest, permanent lawn or dense non-deciduous orchard plantations, remain comparatively invariant throughout the growing season (VPIFs). We have shown drastic differences between the selected VVFs and VPIFs in the spectral bands and vegetation index evolution. The phenotypic or morphological aspects of VPIFs are well known, and therefore, the light reflectance changes very little throughout the annual growing season, which is a key factor for the land use classification of agricultural scenes through remote sensing. Additionally, the pseudo-invariability of VPIF is the characteristic used in the ARIN procedure to normalize a set of images of a common scene.

ARIN method was developed for the radiometric normalization of multitemporal images of agricultural and forestry scenes where vegetative pseudo-invariant features (VPIFs) can be identified. In our work, we have shown that selected mature CIT (citrus orchards), OLI (olive orchards) and POP (poplar groves) in a Mediterranean landscape can be chosen efficiently as VPIFs throughout spring and summer. Generally, the ARIN normalization process efficiently produced relatively uniform data for all images, regardless of the single VPIF or the combination of VPIFs chosen. This result is likely because the range and s. d. of the spectral bands and vegetation indices in the transformed images are considerably reduced compared to those of the original images.

Regardless of the single VPIF used to estimate the band CFs for the image series transformation, the results were relatively normalized when compared to those of the original images. The VPIF spectral band correction factors (CFs) used to implement the ARIN linear normalization procedure varied greatly among spectral bands for any given image and among images for any given spectral band. Furthermore, the high and statistically significant correlation coefficients between the CFs among the VPIFs for any spectral band and for all bands suggest that the ARIN normalization process was efficient regardless of the VPIF selected.

Implementing the ARIN procedure consecutively using two VPIF was also an efficient method of normalizing the multitemporal images series. Generally, for the multitemporal series of GeoEye-1 images studied, ARIN was more efficient than QUAC and as efficient as the FLAASH absolute calibration method. The advantage of the ARIN method is that weather calibration parameters are not necessary, whereas they are required for the highly tunable FLAASH methods.

VPIF size is clearly related to the image spatial resolution and should have a sufficient number of pixels to provide a solid average of the selected vegetative feature. With medium to high spatial resolution images from the satellite GeoEye-1 (i.e., <5 m pixel), VPIF size normally coincides with uniform parcels of approximately 0.3 to 0.5 ha or larger. Moreover, ARIN can also be used with very high spatial resolution images (i. e. 3 to 5 cm pixel) such as those provided by unmanned aerial vehicles (UAV) [26]. In UAV images a VPIF size of 2 to 4 m^2^ will cover a high number of pixels to provide a solid pseudo-invariant vegetative feature sample, and in practice, it may coincide with a non-deciduous tree of 2 to 4 m^2^. UAV images can be normalized through the ARIN procedure avoiding the use of the barium sulfate standard spectralon panel, which is placed in the middle of the field to calibrate data [26].

Step-by-step implementation of the VPIF-based ARIN normalization procedure through the available ENVI image-processing tools is time consuming, and therefore, it is not economically feasible. ARIN software [22] quickly and easily executes ARIN and generates the transformed images; consequently, its development is essential for the practical application of the ARIN method. However ARIN method can be applied only in any agricultural and forestry landscapes where a VPIF can be identified.

## Conclusions

A novel method for the radiometric normalization of multitemporal images, named ARIN, was developed to be used in agricultural and forestry scenes where a vegetative pseudo-invariant features (VPIFs) can be identified. This new procedure identifies one common VPIF parcel in all scenes, extracts the spectral band values of each image and transforms them to common band values through linear transformation. We validated ARIN using a series of GeoEye-1 satellite images of one scene. ARIN worked correctly, regardless of the three VPIFs considered (citrus orchards, olive orchards and poplar groves). The ARIN method was slightly more efficient than the absolute calibration QUAC method and as efficient as the highly tunable FLAASH method, which uses solar position and weather calibration parameters. Implementing the ARIN procedure through conventional image processing is time consuming. The software ARIN executes ARIN semi-automatically in an economically feasible manner.
